# Cost-effectiveness of yoga for managing musculoskeletal conditions in the workplace

**DOI:** 10.1093/occmed/kqx161

**Published:** 2017-11-30

**Authors:** N Hartfiel, G Clarke, J Havenhand, C Phillips, R T Edwards

**Affiliations:** 1 Centre for Health Economics and Medicines Evaluation, Bangor University, Bangor, Wales; 2 School of Healthcare Sciences, Bangor University, Bangor, Wales; 3 Department of Marine Sciences, University of Gothenburg, Gothenburg, Sweden; 4 College of Human and Health Sciences, Swansea University, Swansea, Wales; 5 Centre for Health Economics and Medicines Evaluation, Bangor University, Bangor, Wales

**Keywords:** Back pain, cost-effectiveness, musculoskeletal conditions, occupational health, physical activity, randomized controlled trial, return-on-investment, sickness absence, workplace, yoga

## Abstract

**Background:**

Back pain and musculoskeletal conditions negatively affect the health-related quality of life (HRQL) of employees and generate substantial costs to employers.

**Aims:**

To assess the cost-effectiveness of yoga for managing musculoskeletal conditions.

**Methods:**

A randomized controlled trial evaluated an 8-week yoga programme, with a 6-month follow-up, for National Health Service (NHS) employees. Effectiveness in managing musculoskeletal conditions was assessed using repeated-measures generalized linear modelling for the Roland-Morris Disability Questionnaire (RDQ) and the Keele STarT Back Screening Tool. Cost-effectiveness was determined using area-under-the-curve linear regression for assessing HRQL from healthcare and societal perspectives. The incremental cost per quality-adjusted life year (QALY) was also calculated. Sickness absence was measured using electronic staff records at 6 months.

**Results:**

There were 151 participants. At 6 months, mean differences between groups favouring yoga were observed for RDQ [−0.63 (95% CI, −1.78, 0.48)], Keele STarT [−0.28 (95% CI, −0.97, 0.07)] and HRQL (0.016 QALY gain). From a healthcare perspective, yoga yielded an incremental cost-effectiveness ratio of £2103 per QALY. Given a willingness to pay for an additional QALY of £20 000, the probability of yoga being cost-effective was 95%. From a societal perspective, yoga was the dominant treatment compared with usual care. At 6 months, electronic staff records showed that yoga participants missed a total of 2 working days due to musculoskeletal conditions compared with 43 days for usual care participants.

**Conclusions:**

Yoga for NHS employees may enhance HRQL, reduce disability associated with back pain, lower sickness absence due to musculoskeletal conditions and is likely to be cost-effective.

## Introduction

Musculoskeletal conditions are a common cause of global disability [1]. In UK, musculoskeletal conditions result in >30 million sickness absence days per year [2], costing employers an estimated £5.6 billion [3]. The National Health Service (NHS) is the largest employer in the UK, with ~1.3 million employees. In 2013, 13.7 million days were lost due to NHS sickness absence [4], of which musculoskeletal conditions, primarily related to back pain, accounted for 40% (direct costs of £620 million) [5].

Although few workplace interventions are effective for preventing back pain and musculoskeletal conditions, structured exercise programmes can have a positive effect on the health of employees [6]. The National Institute for Health and Care Excellence (NICE) recommends interventions designed to stretch/strengthen muscles and improve posture [7].

Recent research indicates that short-term yoga programmes (≤12 weeks) can be effective for reducing back pain and musculoskeletal conditions in patient populations [8,9]. Few studies, however, have explored the effectiveness and cost-effectiveness of yoga for relatively healthy employees in workplace settings.

## Methods

A 6-month, multicentre, randomized controlled trial (RCT) was conducted with NHS employees. Ethical approval was obtained from the Bangor University School of Sport, Health and Exercise Sciences, and an NHS Internal Research and Development Review Panel (IRAS #114550).

NHS employees with and without back pain were recruited via an occupational health (OH) e-newsletter and e-mail sent to more than 15000 staff. Participants were stratified by hospital site and gender and ran domized 1:1 to yoga or usual-care. Yoga participants received one free 60-minute session per week for 8 weeks. Sessions were delivered after work at three hospital sites by six instructors who had completed a 200-hour Dru Yoga training course accredited internationally with the Yoga Alliance. Dru Yoga is a style characterized by specific movements, directed breathing, and relaxation methods that include affirmation and visualization techniques. The 60-minute sessions involved four stages: activation exercises, energy block release sequences, back care postures and relaxation techniques. Yoga participants also received a DVD and an illustrated booklet for home practice. Usual-care participants received two evidence-based booklets: *The Back Book* and *How to Manage Stress*. Additional information about the intervention is reported in the study protocol [10].

The effectiveness of the yoga programme was assessed using two valid and reliable measures: the Roland-Morris Disability Questionnaire (RDQ—primary outcome) and the Keele STarT Back Screening Tool (secondary outcome) [11,12]. The RDQ focuses on the loss of physical function, whereas the Keele STarT assesses both physical function and psychosocial factors such as fear, worry, loss of hope and the displeasure associated with back pain.

As described in the study protocol, an *a priori* power calculation determined that 116 NHS participants in total were required for this study [10]. This estimation was based on a pilot study of yoga in the workplace where the standard deviation of the difference in RDQ change scores was 1.95 points. The 1.17 change in RDQ scores from baseline to end programme in this study was found to be statistically significant [6]. A change in RDQ scores between 1 and 2 points can be considered ‘clinically important’ for people with little disability (i.e. employees in workplace settings) [11].

Statistical analysis included all enrolled participants using the Statistical Package in the Social Sciences version 20.0 (SPSS Inc, Chicago, IL, USA). Yoga and usual-care groups were compared at baseline, 8 weeks and 6 months for RDQ and Keele STarT, respectively. Prior to analysis, all data were checked for normality and homogeneity of variance using Q-Q plots and box plots. Between-group differences in RDQ and Keele STarT mean scores were assessed using a repeated-measures analysis of covariance (ANCOVA), a recommended method for analysing baseline and post-treatment measures in RCTs [13]. Missing values for 8-week RDQ scores were imputed from baseline scores using a multiple imputation by chained equations method [14]. This method incorporates multivariable regression techniques to replace missing values with probable substitutes. Substitute values are averaged across a number of replicated datasets equal to the percentage of incomplete cases [15].

Cost-effectiveness was assessed from the healthcare and societal perspectives. The healthcare perspective considered intervention costs and healthcare resource use costs, whereas the societal perspective also included production loss costs.

Intervention costs included yoga mats, cushions, DVDs, illustrated booklets and instructor fees at £60 per session which represented the upper-end range for a typical UK yoga instructor [16]. Healthcare resource use costs were based on self-reported visits to primary care health professionals (e.g. GPs, physiotherapists, osteopaths and massage therapists). Unit costs were obtained from NHS Reference Costs [17] and the Personal Social Services Research Unit [18]. The reference year for pricing was 2013 (reflective of study year) with costs in UK pounds sterling. Production loss costs were calculated from differences in sickness absence days (due to musculoskeletal conditions) between the yoga and usual-care groups using the human capital approach [19]. Costs were monetized using the mean cost per day for an NHS employee (£114 per day) [4]. To account for lower productivity of a substitute worker, the mean cost per day for an NHS employee (£114) was multiplied by 1.28 [20], resulting in an adjusted mean cost per day of £146 per absent worker.

To determine cost-effectiveness, differences in costs between yoga and usual-care groups were compared with differences in health-related quality of life (HRQL) assessed with the EQ-5D-5L at baseline, 8 weeks and 6 months [21]. The EQ-5D-5L measured five domains: mobility, ability to care for oneself, usual activities, pain/discomfort and anxiety/depression. EQ5D-5L scores were then weighted according to a UK value set, and quality-adjusted life years (QALYs) were calculated using two approaches: change from baseline (CfB) and area-under-the-curve (AUC) approach with/without linear regression [22].

Missing values for 6-month EQ-5D-5L scores were imputed from baseline and 8-week scores using a multiple imputation by chained equations method [14]. Using both complete and imputed cases, differences in costs and QALYs between the yoga and usual-care groups were calculated to determine incremental cost-effectiveness ratios:

$$Incrementalcost\;effectiveness\;ratio=\frac{meancost\;yoga\;group-meancost\;self\;care\;group}{mean\;QALY\;yoga\;group\;-mean\;QALY\;self\;care\;group}$$

Due to the uncertainty around costs and effectiveness, bootstrapped incremental cost-effect pairs, using 1000 replications, were plotted on cost-effectiveness planes. Cost-effectiveness planes are graphs with 1000 bootstrap replications comparing incremental gains in HRQL with the incremental costs of the yoga intervention [23]. Cost-effectiveness acceptability curves are graphs summarizing information on uncertainty and in this case estimated the probability that yoga was cost-effective compared with usual-care at a willingness-to-pay threshold of £20 000 per QALY gained [23] (Figure 2).

## Results

**Figure 1.**
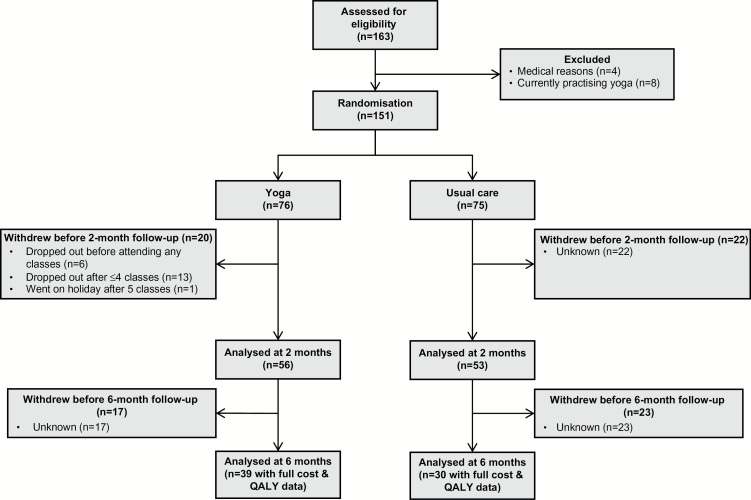
Participant flow diagram.

One hundred and sixty-three employees were recruited. Twelve participants were excluded due to recent spinal disc problems, major surgery, pregnancy or currently practising yoga/yoga-related activities. Eligible employees (*n* = 151) were randomized to yoga (*n* = 76) or usual-care (*n* = 75). The majority of participants were female (93%) with a mean age of 44 years. Approximately half of the participants (51%) had a university degree (bachelors, masters or PhD); 49% had a job profile within NHS bands 4–6 (Table 1). At baseline, 61% of yoga participants and 52% of usual-care participants reported some back pain (non-acute with RDQ ≤ 12). Participant flow is provided in Figure 1.

**Table 1.** Comparison of baseline characteristics between groups

Demographic characteristics	Yoga (*n* = 76)	Usual-care (*n* = 75)
Mean age (y, SD)	44.12 (10.38)	43.60 (11.71)
Gender (*n*, %)	Female = 70 (92) Male = 6 (8)	Female = 70 (93) Male = 5 (7)
Education level	GCE, GCSE, NVQ = 26% Cert/Dip of Higher Ed = 24% Bachelor, Master, PhD = 50%	GCE, GCSE, NVQ = 24% Cert/Dip of Higher Ed = 23% Bachelor, Master, PhD = 53%
NHS band level	Bands 1, 2, 3 = 28% Bands 4, 5, 6 = 47% Bands 7, 8, 9 = 25%	Bands 1, 2, 3 = 28% Bands 4, 5, 6 = 51% Bands 7, 8, 9 = 21%
Back pain at baseline	RDQ = 0 (39%) RDQ = 1–6 (54%) RDQ = 7–12 (7%)	RDQ = 0 (48%) RDQ = 1–6 (44%) RDQ = 7–12 (8%)
RDQ (SD)	2.09 (2.44)	1.93 (2.97)
Keele STarT (SD)	1.37 (1.16)	1.41 (1.40)

GCE, General Certificate of Education; NHS, National Health Service; PhD, doctor of philosophy; SD, standard deviation; RDQ, Roland-Morris Disability Questionnaire.

At 8 weeks, 56 (74%) yoga participants and 53 (70%) usual-care participants completed end-programme questionnaires. The average yoga participant attended six classes during the 8-week programme and practised at home for 60 minutes per week. Repeated measures ANCOVA (complete case analysis) showed that when compared with usual-care, yoga was associated with statistically significant reductions in back pain-related disability: RDQ [−0.84 (95% CI −1.78, −0.06); *p* < 0.05] and Keele STarT [−0.61 (95% CI −1.19, −0.39); *p* < 0.001] (Table 2).

**Table 2.** Mean scores (SD), mean differences, confidence intervals and p-values

	Roland-Morris Disability Questionnaire (RDQ)		Keele STarT Back Screening Tool	
	Yoga	Usual care	Yoga	Usual care
Baseline all cases	2.09 (2.44) *n* = 76	1.93 (2.97) *n* = 75	1.37 (1.16) *n* = 76	1.41 (1.40) *n* = 74
Baseline complete cases	2.05 (2.33) *n* = 56	2.23 (3.12) *n* = 53	1.32 (1.03) *n* = 56	1.57 (1.39) *n* = 53
End programme at 8 weeks	1.34 (1.72) *n* = 56	2.36 (3.44) *n* = 53	0.76 (0.77) *n* = 55	1.62 (1.36) *n* = 53
Mean change at 8 weeks	–0.71	0.13	–0.56	0.05
Mean difference between groups at 8 weeks [95% CI], *P*-value^a^	–0.84 [–1.78,–0.06], *P* < 0.05		–0.61 [–1.78,0.48], *P* < 0.001	
Follow-up at 6 months	1.26 (2.05) *n* = 43	2.03 (3.30) *n* = 32	0.95 (1.17) *n* = 42	1.50 (1.30) *n* = 32
Mean change at 6 months	–0.79	–0.20	–0.37	–0.07
Mean difference between groups at 6 months [95% CI], *P*-value	–0.59 [–1.78,0.48], *P* = NS		–0.30 [–0.97,0.07], *P* = NS	

aUnadjusted *P*-values for multiple comparisons.

At 8 weeks, end-programme questionnaires were not completed by 20 yoga participants and 22 usual-care participants, resulting in 28% incomplete cases. Of the 20 yoga participants, six never turned-up and 14 attended at least one session. Reasons for withdrawal included childcare commitments (*n* = 3), adverse events unrelated to yoga (*n* = 2), holiday (*n* = 1), adverse events related to yoga (*n* = 1, mild muscle spasm) and unknown (*n* = 7). To deal with missing data, 28 imputed datasets were created; 82% resulted in statistically significant differences in RDQ between yoga and usual-care. Data from imputed cases generated a slightly greater intervention effect (−0.99) than complete cases (−0.84).

At 6 months, the difference in RDQ mean scores between groups was no longer statistically significant due to a smaller effect size (mean RDQ between-group difference was −0.84 at 8 weeks and −0.63 at 6 months) and greater variance between groups (between-group 95% CI for mean RDQ differences at 8 weeks was −1.78 to −0.06 compared with −1.78 to +0.48 at 6 months). In addition, the high percentage of incomplete cases at 6 months (54%) may have reduced the statistical power necessary to detect meaningful differences between groups. Nevertheless, these results suggest that the long-term benefits of yoga may depend on the continuation of weekly classes. At 6 months, 22% of the yoga participants were no longer practising, 68% were practising ‘sometimes’ or ‘once per week’ and 10% were practising ‘three times or more per week’.

The cost-effectiveness analysis included 39 (51%) yoga participants and 30 (40%) usual-care participants who completed 6-month follow-up questionnaires (Figure 1). Although 82 non-completers (54%) were e-mailed twice regarding the 6-month questionnaire, OH staff did not have time for additional follow-up due to an unexpected demand for NHS staff influenza vaccinations.

To estimate the cost-effectiveness of yoga, QALY gains were compared with total costs between groups. Using complete case analysis, yoga participants reported QALY gains of 0.047 (CfB method), 0.034 (AUC method) and 0.027 (AUC with linear regression) (Table 3). Using imputed case analysis for 54 imputed datasets, yoga participants reported QALY gains of 0.033 (CfB method), 0.017 (AUC method) and 0.016 (AUC with linear regression) (Table 3). Ordinary least squares regression was used to control for baseline differences. Bias-corrected and accelerated bootstrapping was performed using 1000 replicates to calculate non-parametric 95% confidence intervals.

**Table 3.** EQ-5D-5L index scores (SE): 6-month QALYs, complete and imputed cases

Measure EQ-5D-5L (complete cases)	Yoga	Usual-care	Difference in mean scores from baseline	Difference in mean scores between groups^a^
Complete cases	*n* = 39	*n* = 30		
Baseline End-programme 6 months	0.836 (0.017) 0.857 (0.017) 0.850 (0.018)	0.815 (0.016) 0.776 (0.022) 0.782 (0.023)	0.081 0.068	0.021 0.060 (8 weeks—CfB) 0.047 (6 months—CfB)
6 month (AUC) [95% CI] Multiple linear regression [95% CI]	0.426 0.450	0.392 0.423		0.034 (6 month—AUC w/o regression) [0.010, 0.056] 0.027 (6 month—AUC with regression) [0.009, 0.046]
Imputed cases	*n* = 2106	*n* = 1620		
Baseline End-programme 6 months	0.839 (0.012) 0.846 (0.014) 0.844 (0.018)	0.838 (0.012) 0.802 (0.016) 0.811 (0.020)	0.044 0.033	0.001 0.043 (8 weeks—CfB) 0.032 (6 months—CfB)
6 months (AUC) [95% CI] Multiple linear regression [95% CI]	0.422 0.438	0.405 0.422		0.017 (6 months—AUC w/o regression) [0.015, 0.019] 0.016 (6 months—AUC with regression) [0.014, 0.018]

AUC, area-under-curve; CfB, change from baseline; CI, confidence interval; SE, standard error; w/o, without.

^a^Accounting for baseline differences.

Total costs were comprised of intervention costs, healthcare resource use costs and production loss costs (from sickness absence). Intervention costs for yoga participants (equipment costs + instruction costs) were £56.52 per person compared with £2.00 per person for usual-care (cost of evidence-based booklets) (Table 4). Total equipment costs for yoga participants were £1416 (36 yoga mats = £360; 36 cushions = £144; 76 DVDs = £912) and mean equipment costs were £18.63 per person. Total instruction costs for yoga were £2880 (48 sessions at £60 per session) and mean instruction costs were £37.89 per person.

Healthcare resource use costs (back pain and musculoskeletal-related conditions) were £5.87 per yoga participant versus £26.73 per usual-care participant (Table 4). During the 6-month study, complete case analysis of self-reported questionnaires showed that yoga participants made five visits (0.13 visits per person) to primary care health professionals for back pain and musculoskeletal conditions. This compared with 18 visits (0.60 visits per person) made by usual-care participants.

Production loss costs were £4.07 per person for yoga and £92.49 per person for usual-care—a difference of £88.42 per person when using complete case data based on the £114 mean cost per day for an NHS employee (Table 4). When extra sickness absence-related costs were applied (multiplier of 1.28), the difference between groups was £113.18 per person (Table 4). The difference in production loss costs between groups can be explained from employee staff records at 6 months which indicated that yoga participants who attended at least one class (*n* = 56) missed a total of 2 days due to musculoskeletal conditions compared with a total of 43 days missed by for usual-care participants (*n* = 53).

**Table 4.** Healthcare and societal perspectives: differences in costs and QALYs

	Healthcare perspective	Societal perspective
Intervention costs		
Yoga total	**£56.52/person**	**£56.52/person**
Equipment cost	£18.63/person	£18.63/person
Instruction cost	£37.89/person	£37.89/person
Usual-care total	**£2.00/person**	**£2.00/person**
Difference in total intervention costs between groups: £54.52/person		
Healthcare resource use costs (based on participant self-report at 8 weeks/6 months)^a^		
Yoga (*n* = 39)		
Total cost	£229	£229
Visits/person^b^ (CI)	0.13 (−0.02 to 0.28)	0.13 (−0.02 to 0.28)
Cost/person (CI)	**£5.87** (£−0.76 to £12.50)	**£5.87** (£−0.76 to £12.50)
Usual-care (*n* = 30)		
Total cost	£802	£802
Visits/person^b^ (CI)	0.60 (0.02 to 1.18)	0.60 (0.02 to 1.18)
Cost/person (CI)	**£26.73** (£1.18 to £52.49)	**£26.73** (£1.18 to £52.49)
Production loss costs (based on electronic staff records)		
Yoga (*n* = 56)		
Total cost		
Missed days/person (CI)	N/A	£228
Cost/person (CI)	N/A	0.04 (−0.03 to 0.11)
Usual-care (*n* = 53)	N/A	**£4.07** (−£3.91to £12.05)
Total cost	N/A	£4,902
Missed days/person (CI)	N/A	0.81 (−0.29 to 1.91)
Cost/person (CI)	N/A	**£92.49** (−32.77 to £217.75)
Difference in sickness absence costs between groups (difference: 4 participants, 41 days)	N/A	£4,674 total cost **£88.42/person**
Difference in sickness absence costs between groups with 1.28 multiplier (difference: 4 participants, 41 days)	N/A	£5,983 total cost **£113.18/person**
Total cost		
Yoga	**£62.49/person**	**£66.56/person**
Usual-care	**£28.73/person**	**£121.22/person**
Difference in costs between groups	**£33.76/person**	**−£54.66/person**
Bootstrapped 95% CI	−£8 to £56	−£389 to £32
QALYs (complete cases)	0.027	0.027
Bootstrapped 95% CI	−0.003 to 0.057	−0.003 to 0.057
QALYs (imputed cases)	0.016	0.016
Cost/QALY (ICER—complete cases)	£1,246	Yoga dominant
Cost/QALY (ICER—imputed cases)	£2,103	Yoga dominant
Cost-effectiveness probability—complete cases (£20000/QALY threshold)	95%	98%

CI, confidence interval; ICER, incremental cost-effectiveness ratio; N/A, not applicable; QALY, quality adjusted life year; SD, standard deviation. Bold entries are total costs per person.

^a^Number of visits to health professionals in primary care settings during 6-month study.

^b^GP unit cost £53 (Curtis, L. 2013. Unit costs of health and social care. University of Kent: Personal Social Services Unit).

Yoga = £53 total, £1.36/person (visits baseline to 8 weeks = 0; visits 8 weeks to 6 months = 1, total visits = 1).

Usual-care = £159 total, £5.30/person (visits baseline to 8 weeks = 1; visits 8 weeks to 6 months = 2, total visits = 3).

^b^Physiotherapist unit cost £44 (Department of Health. 2013. Reference costs 2012–13).

Yoga = £176 total, £4.51/person (visits baseline to 8 weeks = 0; visits 8 weeks to 6 months = 4, total visits = 4).

Usual-care = £132 total, £4.40/person (visits baseline to 8 weeks = 1; visits 8 weeks to 6 months = 2, total visits = 3).

^b^Osteopath unit cost £43 (http://www.osteopathy.org.uk/visiting-an-osteopath/what-to-expect/).

Yoga = £0 (visits baseline to 8 weeks = 0; visits 8 weeks to 6 months = 0, total visits = 0).

Usual-care = £473 total, £15.77/person (visits baseline to 8 weeks = 7; visits 8 weeks to 6 months = 4, total visits = 11).

^b^Massage therapist unit cost £38 (https://nationalcareersservice.direct.gov.uk/advice/planning/jobprofiles/Pages/massagetherapist.aspx).

Yoga = £0 (visits baseline to 8 weeks = 0; visits 8 weeks to 6 months = 0, total visits = 0).

Usual-care = £38 total, £1.27/person (visits baseline to 8 weeks = 0; visits 8 weeks to 6 months = 1, total visits = 1).

**Figure 2.**
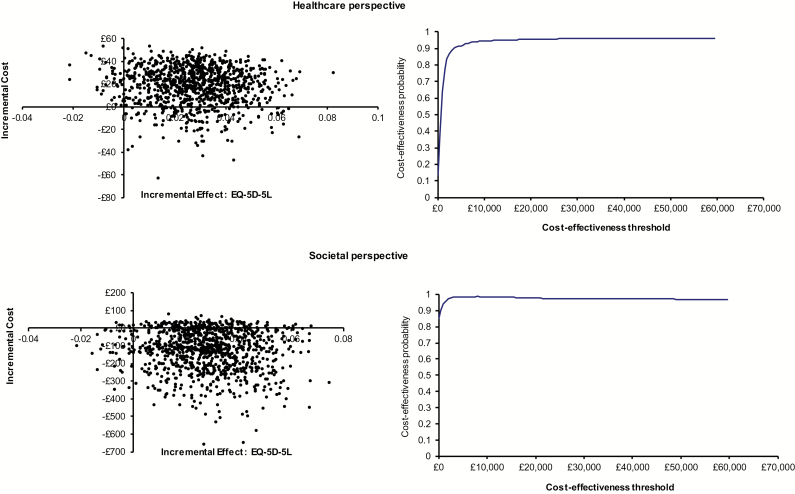
Healthcare and societal perspectives: cost-effectiveness planes and cost-effectiveness acceptability curves.

From the healthcare perspective (intervention costs + healthcare resource use costs), total costs were £62.49 per yoga participant compared with £28.73 per usual-care participant. The cost-effectiveness plane indicated that most replicated cost-effect pairs (91%) fell in the north-east quadrant, indicating that yoga was more effective and more costly than usual-care (Figure 2). The incremental cost-effectiveness ratios ranged from £1246 (complete cases) to £2103 (imputed cases), well below the $20 000 QALY threshold.

From the societal perspective (intervention costs + healthcare resource use costs + production loss costs), total costs were £66.46 per yoga participant versus £121.22 per usual-care participant (Table 4). The cost-effectiveness plane showed most replicated cost-effect pairs (81%) in the south-east quadrant indicating that yoga was *dominant* to usual-care (i.e. more effective and less costly) (Figure 2).

Using complete case analysis, the cost-effectiveness acceptability curves showed that from healthcare and societal perspectives, the probability of yoga being cost-effective versus usual-care at the £20 000 QALY threshold was 95% and 98%, respectively (Table 4, Figure 2).

## Discussion

Compared with usual care, yoga participants reported greater reductions in back pain-related disability at 8 weeks and 6 months. The difference between groups indicated that yoga addressed both the physical and psychological components of back pain. For RDQ, however, the mean difference between the two groups was less than 1 point, suggesting a non-clinically im portant difference for this relatively healthy employee population.

At 6 months, yoga was associated with a mean QALY gain of 0.016 using an AUC linear regression analysis for imputed cases [22]. In a previous cost-effectiveness study of yoga for a patient population, yoga participants reported a mean QALY gain of 0.037 for imputed cases [24]. The difference in QALY gains between these two studies may be due to the relatively healthy employee population used in this study where the mean EQ-5D score at baseline was 0.826 compared with a mean EQ-5D score of 0.705 in the previous study [24]. In addition, the time frame for this study was 6 months compared with 12 months in the previous study [24]. Despite these differences in target population and length of study, findings from both studies suggest that yoga is associated with improved HRQL.

From the healthcare perspective, yoga was more costly than usual-care due to the cost of implementing the yoga programme. Intervention costs for yoga participants were £54.52 per person more than for usual-care participants. However, healthcare resource use costs for yoga were £20.86 less per person than for usual-care. This is consistent with other studies indicating that yoga is associated with reduced healthcare resource use costs [25].

Observed sickness absence related to back pain and musculoskeletal conditions in this study was also consistent with the results of previous studies of yoga for patients with low back pain [24,26]. When compared with usual-care, yoga participants in these previous studies reported 8.5 and 17.2 fewer sickness absence days per person per year (due to back pain) [24,26]. These findings suggest that yoga may reduce sickness absence due to back pain and musculoskeletal conditions, potentially representing cost-savings for employers.

Although the cost-effectiveness of yoga in this study appears promising, the results need to be interpreted with caution due to a number of limitations, including the small sample size. During the 8-week programme and 6-month follow-up, only six participants missed work due to musculoskeletal conditions including back pain (usual-care: *n* = 5; yoga: *n* = 1). Of the five usual-care participants, one missed 29 days, accounting for 67% of the total. Although it may be argued that yoga could have prevented these 29 days of sickness absence, this outlier may have overinflated cost savings attributed to yoga.

The amount of missing data at 6 months (54%) could also raise concerns about attrition bias. Non-completion is often a problem with physical activity interventions in the workplace, where more than 50% of participants commonly drop out at some point [27]. In yoga trials, there is a clear association between study length and dropout rate, with almost double the attrition in studies lasting >12 weeks [28].

The 54% attrition rate at 6 months could be attributed to the response burden, lack of financial incentives and absence of telephone follow-up with participants. Ethical approval of this study required that communication with study participants occurs through OH staff. At 6 months, OH staff sent two reminder emails to non-completers (54%) regarding completing follow-up questionnaires. However, due to work commitments, OH staff had insufficient time to make any further contact with non-completers.

To assess the degree of attrition bias, baseline RDQ characteristics of completers and non-completers at 8 weeks and 6 months were compared. No significant differences in baseline characteristics were found using independent samples *t*-tests at each time point. It, therefore, appears probable that attrition bias was minimized due to the similarity between completers and non-completers.

Multiple imputation was also used to help overcome potential biases due to missing data. Complete case and imputed data were compared, and the results for RDQ and HRQL outcomes were similar for both. Using imputed cases, the intervention effect was slightly larger for RDQ and smaller for HRQL.

Presenteeism costs, overhead costs and opportunity costs were also not included in this study. Presenteeism costs were not included in this study due to difficulties in measurement and lack of consensus regarding their inclusion. Overhead costs were negligible as no additional heating was required, and electricity was used only for lighting. Opportunity costs were minimal given that yoga classes were voluntary, occurred after work and participants reported high satisfaction.

Finally, participants in this study were self-selected, and therefore representative of employees interested in workplace yoga. In addition, improvements for yoga participants may have been caused by other factors such as instructor influence or group participation.

Strengths of this study include the multi-centre, randomized, controlled design. The use of six instructors at three sites compares favourably with most yoga trials which include one instructor at one location [29]. NHS employee staff records provided a more accurate measure of sickness absence than self-report [30]. Employees with and without back pain were included, indicating that yoga may be effective in preventing, as well as treating, back pain.

This study indicated that an 8-week Dru Yoga programme, compared with usual-care, was associated with improvements in health-related quality of life and reductions in both physical and psychosocial components of back pain. Yoga in the workplace appeared to be a cost-effective option, potentially reducing sickness absence due to musculoskeletal conditions. Economic evaluation alongside larger RCTs are needed to further explore the efficiency of yoga as a workplace health intervention.

**Key points**

- This study is the first to evaluate the cost- effectiveness of yoga for managing musculoskeletal conditions in an employee population.
- Compared with usual care, the probability of yoga being cost-effective for an employee population was 95% at a willingness-to-pay threshold of £20,000 per QALY.
- Yoga for employees may improve health-related quality of life, reduce disability associated with back pain and provide a potentially cost-effective option for employers.

## Conflicts of interest

None declared.
